# Mechanism of Cross-Species Transmission, Adaptive Evolution and Pathogenesis of Hepatitis E Virus

**DOI:** 10.3390/v13050909

**Published:** 2021-05-14

**Authors:** Putu Prathiwi Primadharsini, Shigeo Nagashima, Hiroaki Okamoto

**Affiliations:** Division of Virology, Department of Infection and Immunity, Jichi Medical University School of Medicine, Tochigi 329-0498, Japan; thiwik8@jichi.ac.jp (P.P.P.); shigeon@jichi.ac.jp (S.N.)

**Keywords:** hepatitis E virus, pathogenesis, cross-species transmission, ORF1, codon usage, adaptive evolution, host cellular factor

## Abstract

Hepatitis E virus (HEV) is the leading cause of acute hepatitis worldwide. While the transmission in developing countries is dominated by fecal-oral route via drinking contaminated water, the zoonotic transmission is the major route of HEV infection in industrialized countries. The discovery of new HEV strains in a growing number of animal species poses a risk to zoonotic infection. However, the exact mechanism and the determinant factors of zoonotic infection are not completely understood. This review will discuss the current knowledge on the mechanism of cross-species transmission of HEV infection, including viral determinants, such as the open reading frames (ORFs), codon usage and adaptive evolution, as well as host determinants, such as host cellular factors and the host immune status, which possibly play pivotal roles during this event. The pathogenesis of hepatitis E infection will be briefly discussed, including the special forms of this disease, including extrahepatic manifestations, chronic infection, and fulminant hepatitis in pregnant women.

## 1. Introduction

Hepatitis E virus (HEV) is a single-stranded positive-sense RNA virus that belongs to the family *Hepeviridae* [1]. HEV is the leading cause of acute hepatitis around the world. The World Health Organization (WHO) estimated that there are 20 million HEV infections worldwide with approximately 44,000 deaths in 2015 [2]. HEV is known to cause acute hepatitis, rarely leading to fulminant hepatitis. However, chronic hepatitis has increasingly been reported in immunocompromised patients, including organ transplant recipients, as well as patients with hematological malignancy and human immunodeficiency virus (HIV) infection [3,4,5]. Besides manifesting as typical hepatitis, HEV infection can also cause extrahepatic manifestations, including neurological manifestations [6,7], kidney injury, and hematological disorders [8,9].

Among the five known hepatitis viruses, HEV has unique transmission routes ranging from the fecal-oral route, as its classical mode of transmission, and the zoonotic route, to less frequent routes such as via organ transplantation, transfusion of blood or blood products, or vertical transmission from mother to fetus [3,10,11,12,13,14]. Although the overall mortality rate is low, it could reach as high as 30% in infected pregnant women entering their third trimester of pregnancy [14].

HEV infection is globally distributed. In developing countries, HEV has caused multiple outbreaks where the virus is transmitted via the fecal-oral route by drinking contaminated water. Meanwhile, HEV infections in industrialized countries are mainly associated with zoonotic causes [15]. The isolation of the first HEV strain in an animal, in swine [16], opened the door to the discovery of more HEV strains and their variants in animal species. The report by the same research group in the following year showed evidence that some HEV strains recovered from patients with acute hepatitis E are closely related to swine HEV, which can cross the species barrier to infect humans [17,18], proving an HEV zoonosis event. 

Isolation of new HEV strains in a growing number of animal species poses a risk to zoonotic infection and has become a public health concern. Despite the inevitable risk of zoonotic infection, many questions surrounding the mechanism of the cross-species transmission in HEV infection remain unanswered and will be discussed in this review, encompassing the viral and host factors involved in the event, such as the viral adaptive evolution and host cellular factors, as some of the determinants of host species tropism. In addition, the pathogenesis of hepatitis E infection will be briefly described in this review, including special forms of the disease, such as chronic infection, extrahepatic manifestations, and the severe cases in pregnant women.

## 2. Genome and Classification

**Figure 1:**
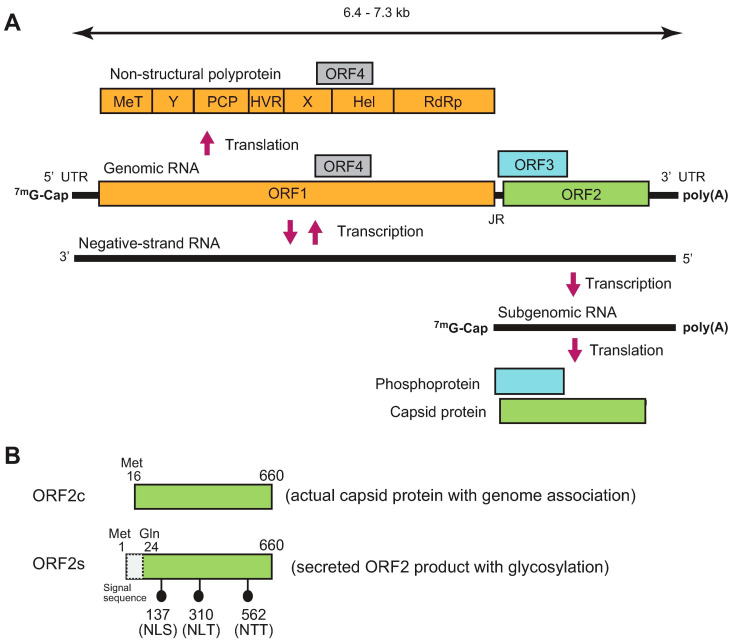
The genomic organization and translation of HEV. (**A**) Genome map of HEV. MeT, Methyltransferase; Y, Y domain; PCP, Papain-like cysteine protease; HVR, Hypervariable region; X, macro domain; Hel, Helicase; RdRp, RNA-dependent RNA polymerase; JR, junction region having the stem-loop structure and playing a critical role in HEV replication [19,20,21,26]. (**B**) A schematic representation of two major forms of ORF2 protein. The actual capsid protein (ORF2c) is initiated from an internal AUG (Met 16) located 16 codons downstream of the first AUG, while the secreted form of the ORF2 product (ORF2s) is initiated from the previously presumed start codon (Met 1) and its N-terminal 23 amino acids are cleaved by signal peptidase [30].

HEV is a single-stranded positive-sense RNA virus. Its genome ranges from 6.4 kilobases (kb) to 7.3 kb with three major open reading frames (ORFs; ORF1–ORF3) (Figure 1A). The genome has a short 5′ untranslated region (5′ UTR) capped at its 5′ end and a short 3′ UTR terminated by the poly(A) tract [19,20]. ORF1 is translated from genomic RNA, while ORF2 and ORF3 are translated from a subgenomic RNA strand [21,22]. ORF1 covers two-thirds of the genome, and encodes a non-structural polyprotein containing multiple functional domains involved in viral replication. However, whether the ORF1 product functions as a single polyprotein or whether it needs to be further processed into smaller units by viral or cellular proteases following translation remains to be elucidated [23,24]. Notably, however, in a recent hemagglutinin (HA)-tagged full-length HEV replicon system, only an uncleaved ~190 kDa ORF1 product was detected [25]. ORF2 encodes capsid protein, which is essential during virion assembly and viral attachment to host cells, and is a major target for neutralizing antibodies [26,27,28]. Recently, it has been reported that HEV produces three forms of ORF2 capsid proteins: the infectious/intracellular ORF2 (ORF2i), which is associated with infectious particles, as well as glycosylated ORF2 (ORF2g) and cleaved ORF2 (ORF2c), which are not associated with infectious particles [29]. Another study determined the initiation codons for the secreted form of the ORF2 product and the actual capsid protein [30]. The secreted form of the ORF2 product (ORF2s) is initiated from the previously presumed start codon (Met 1) and its N-terminal 23 amino acids are cleaved by signal peptidase, while the actual capsid protein (ORF2c) is initiated from an internal AUG (Met 16) located 16 codons downstream of the first AUG (Figure 1B). Between the two studies, functionally, the ORF2s and ORF2c may correspond to ORF2g and ORF2i, respectively, with the absence of the cleaved form ORF2c, presumably due to its lower level [29]. ORF3 encodes a multifunctional phosphoprotein required for virion egress [31,32,33] and has been reported to be a functional ion channel, as viroporin [34].

Exclusively in genotype 1 HEV (HEV-1), ORF4 codes for a novel protein identified in the coding sequence of ORF1. It is synthesized only under the condition of endoplasmic reticulum (ER) stress and is a short-lived protein. ORF4 protein is reported to enhance the replication of HEV-1 by promoting the viral RNA-dependent RNA polymerase (RdRp) activity of ORF1 [35]. A recent report demonstrated that ORF4 provided *in trans* enhances the viral replication of genotype 3 HEV (HEV-3) although it does not naturally encode ORF4 in its genome [36]. 

HEV is a member of family *Hepeviridae* [1], which has been identified in humans as well as a broad range of animal species [15]. This family is divided into genus *Orthohepevirus* which consists of HEV strains from mammals and birds, and genus *Piscihepevirus* with its single species, *Piscihepevirus A*. The genus *Orthohepevirus* consists of species *Orthohepevirus A*, *Orthohepevirus B*, *Orthohepevirus C*, and *Orthohepevirus D*. To date, there are eight genotypes within *Orthohepevirus A* species which infect humans, pigs, wild boars, rabbits, deer, camels, and other animal species. Species *Orthohepevirus B* is only identified in birds, species *Orthohepevirus C* is identified in rodents, while species *Orthohepevirus D* is identified in bats [1]. 

*Orthohepevirus A* has been assigned to eight distinct genotypes (HEV-1–8) [1]. While HEV-1 and HEV-2 have only been isolated in humans, HEV-3 and HEV-4 have been identified in humans as well as several animal species, including pigs, wild boars, deer, and rabbits [37,38,39,40,41,42]. HEV-5 and HEV-6 have only been detected from wild boars in Japan [43,44,45,46]. HEV-7 has been isolated in dromedary camels and a human [47,48,49]. Finally, HEV-8 has been detected in Bactrian camels [50,51]. In addition, there are many as-yet unclassified HEV strains identified from moose [52,53], tree shrew (GenBank accession number KR905549), sparrow [54], silkie fowl [55], little egret [56], hamster [57], voles [58,59], and various bats [60]. 

## 3. Modes of Transmission

In developing countries, HEV is mainly transmitted via the fecal–oral route and has been associated with multiple outbreaks due to poor sanitary conditions (e.g., contaminated drinking water). Meanwhile, sporadic cases and clusters in industrialized countries are often reported where the routes of transmission are more variable and are dominated by zoonotic infection [6,61,62,63,64,65]. Although less frequent than the two main transmission routes, HEV can also be transmitted through organ transplantation or the transfusion of blood or blood products where it can cause chronic HEV infection or from an infected mother to the fetus [3,10,11,12,13,14]. 

Zoonotic HEV is mainly transmitted via foodborne routes, such as from the consumption of undercooked meat or milk products of infected animals. Another possible route is associated with occupational risk among workers, including veterinarians, forestry workers, slaughterhouse workers, or animal farm workers [66,67,68] as they are continuously in direct contact with probably infected animals. 

HEV is excreted in feces where the viral particles can reach the environment and contaminate water sources and irrigated food. HEV in environmental samples can reach the food chain (e.g., in mussels and contaminated pork products, such as sausages or meat) [69]. Besides being detected in packaged sausages and liver samples from processing sites and supermarkets in Spain, Italy, and Czech Republic, HEV is also detected from environmental samples collected in production farms and processing plants and at the points of sale of items such as knives, flooring, belt surfaces, workers’ hands, and toilets [69,70]. Foods other than pork products have also been implicated, where HEV is isolated from shellfish, fruits, and vegetables [71]. This is likely due to pig slurry contaminating watercourses or being used as fertilizer [72]. Coastal water may be contaminated with HEV, leading to the accumulation of the virus in the digestive tissue of shellfish due to filter-feeding [69]. An outbreak of hepatitis E on a cruise ship was linked to the consumption of shellfish while on board [73]. HEV has also been detected in sewage and run-offs, where the virus is concentrated in waste products generated during sewage and drinking water treatment. The following land application of the waste can cause the contamination of water in aquifers, can contaminate irrigated vegetables, and can therefore be a risk factor for HEV infection [69,74,75,76]. Another transmission route that warrants further exploration is through contact with an environment contaminated with the droppings or body fluid of infected animals. 

## 4. Pathogenesis 

The HEV particles in bile and those shed in the feces are non-enveloped. Meanwhile, the particles in circulating blood and culture supernatants are in membrane-associated form, which is different from classical enveloped viruses due to the absence of viral glycoproteins in the surrounding lipid bilayer in which membrane-associated HEV particles seem to be completely covered with a lipid membrane [31,77,78]. These findings have led to the designation of the membrane-associated form of HEV as quasi-enveloped HEV [79,80]. Although a primary site of HEV propagation in humans remains undetermined, it is tempting to speculate that HEV replicates primarily in the liver (not in the gut) and that the non-enveloped virions entering via the gastrointestinal tract will be neutralized by immune sera before reaching the liver via the portal vein. This can explain how humans who were immunized with a recombinant ORF2-based vaccine can be protected against infection with enterically-transmitted HEV [78,81].

Previously, it was hypothesized that lipid-associated HEV is released from both infected cultured cells (in vitro) and infected hepatocytes (in vivo) as lipid-associated virions, accompanied by ORF3 protein, and that the quasi-envelope is dissociated from the virion after shedding the lipid in the bile duct, which contains detergent (deoxycholic acid), and shedding the ORF3 protein in the duodenum, which contains protease (trypsin) secreted from the pancreas [82,83,84]. In support of this hypothesis, Capelli et al. [85] utilized F2 cells (subclone of the human hepatocarcinoma cell line HepG2/C3A, which grew as a polarized monolayer culture and had better HEV production than the mother cell line) in a cell culture due to the polarized nature of hepatocytes, which have exosomal pathways (basolateral, oriented toward the blood; and apical, oriented toward the bile). In this study, they showed that irrespective of the virus form (naked or membrane-associated), and whether HEV-3 or HEV-1 is used as inoculum, the polarized cells released around 95% of the HEV RNA from their apical sides as lipid-associated particles. The ratios of infectious particles to HEV RNA copies on the apical were higher in comparison to the basolateral side, suggesting that the apical pathway is the main release route and that the majority of infectious HEV particles are released from the bile sides of hepatocytes while the small fraction released into the bloodstream could spread HEV throughout the host [85].

Although most HEV infections are self-limiting, infections in immunosuppressed patients may progress into chronic disease or may cause extrahepatic manifestations [6,7,8,9,86]. The host innate immune system is the first line of defense against viral infection. Dysregulation of this system can cause severe pathogenesis. In HEV infection, host innate immunity has shown to involve an active response in experimental models and patients [87,88,89,90,91]. HEV has also developed strategies to counteract the host immune system where recent knowledge mainly outlines the interactions between HEV viral proteins with the host innate immunity, as reported for ORF1 (e.g., the papain-like cysteine protease (PCP) and X domains of ORF1 inhibit the activation of retinoic acid-inducible gene I (RIG-I) and TANK-binding-kinase 1 (TBK-1), as well as the phosphorylation of interferon regulatory factor 3 (IRF3)) [92], ORF2 (e.g., ORF2 suppresses nuclear factor-kappa B (NF-κB) activity by blocking the ubiquitination of inhibitor (IκBα) of NF-κB alpha) [93], and ORF3 (e.g., ORF3 inhibits the phosphorylation of signal transducer and activator of transcription 1 (STAT1) and the expression of interferon-stimulated gene (ISG) upon HEV infection) [94].

Extrahepatic manifestations reported in HEV-infected patients are possibly associated with either direct viral effects due to cytopathic tissue damage caused by extrahepatic replication in affected tissues, or with indirect effects related to the immunological processes induced by a cross-reactive host immune response to HEV [95]. Besides being detected in various tissues in vivo, the ability of HEV to cause extrahepatic manifestations has also been demonstrated in vitro, where HEV can infect a range of cell types (e.g., human lung epithelial cells, human colon epithelial cells, human neuronal-derived cells, and human placental cells) [95,96]. 

HEV infection is associated with a high mortality rate in pregnant women, particularly those in their third trimester of pregnancy, where the highest incidence is reported for HEV-1 [14,97,98]. The alteration of the immune system during hormonal changes in pregnancy has been reported as a potential pathogenic mechanism of HEV infection, which is related to cellular immunity [14,99]. A recent study by Gong et al. [100] indicated that HEV infection significantly inhibits the signaling pathways of cyclic adenosine 3′, 5′-monophosphate/protein kinase A/response element-binding protein (cAMP/PKA/CREB) and phosphatidylinositol 3-kinase/protein kinase B/mammalian target of the rapamycin (PI3K/AKT/mTOR). The increasing estrogen levels and high activation of estrogen receptor alpha (ER-α) during pregnancy aggravates HEV replication. The exacerbation of HEV replication inhibits the expression of ER-α and suppresses the cAMP/PKA/CREB and PI3K/AKT/mTOR signaling pathways [100]. Another factor that might enhance the virulence of HEV in pregnant women is the presence of the ORF4 protein, which is exclusively found in HEV-1. The ORF4 protein is only synthesized under the condition of ER stress and is essential to stimulate the RdRp of HEV-1, which causes enhanced virus replication [35]. Since pregnancy can induce ER stress and fulminant hepatitis E in pregnant women is mostly caused by HEV-1, it can be hypothesized that ER stress caused by pregnancy can induce the synthesis of ORF4, thereby causing higher viral replication (in comparison to non-pregnant patients) and consequently leading to the development of fulminant HEV infection. 

Most chronic HEV infections are associated with HEV-3, with a single case of HEV-1 [101] or HEV-7 [49], which to date, are only anecdotal and unique; and HEV-4, which has been described in small numbers of reports [102]. Viral and host factors are likely the determinants of the chronicity of HEV infection [103]. Viral factors, such as genotype, zoonotic potential, specificity, and adaptability to a host play important roles in the establishment of persistent infection. Meanwhile, host factors, such as the immune state and the nutritional health of the patient can also determine the course of HEV infection. The type of immunosuppressive drugs is reported to affect the progression to chronic infection, with patients treated with calcineurin inhibitors (e.g., tacrolimus, cyclosporin A) or mTOR inhibitors (e.g., everolimus, sirolimus), reported to be more prone to persistent HEV infection [3,103]. 

## 5. Zoonotic HEV Infection

HEV infection caused by zoonotic HEV-3 or HEV-4 has increased in industrial countries, including Japan and several European countries such as France, the Netherlands, Spain, and UK [69,104,105,106]. It is also found to be endemic in the US population and circulates abundantly among North American pig herds [37,106]. Zoonotic transmission is responsible for the sporadic cases and clusters of human infection caused by HEV-3 and HEV-4, and chronic infection reported in immunocompromised patients is mostly linked to zoonotic HEV-3 [37]. 

Swine are the primary reservoir of HEV; in swine, infection is subclinical [107]. Most animal strains of HEV have been isolated from swine. Soon after the report of the first known zoonotic infection related to HEV [17], the number of zoonotic HEV infections continued to increase, particularly in industrialized countries. Within species *Orthohepevirus A*, HEV strains of five genotypes (HEV-1, -2, -3, -4, and -7) are capable of infecting humans (Table 1). While HEV-1 and HEV-2 are restricted to humans, HEV-3, -4, and -7 are capable of crossing the species barrier to cause zoonotic infection in humans. Besides HEV-3 and HEV-4 strains from pigs, wild boars, and deer, variants of HEV-3 in rabbits were also found to be capable of causing human infection [42,108].

Under experimental conditions, swine HEV-3 and HEV-4 infected rhesus monkeys and, conversely, HEV-3 and HEV-4 of human origin infected pigs [17,109,110]. However, HEV-1 and HEV-2 failed to infect pigs under experimental conditions [18], indicating that HEV-1 and HEV-2 have a limited host range [111].

To date, only a single case of human infection related to HEV-7 has been reported. It is a chronic case in an immunocompromised patient caused by the regular consumption of milk and meat products of dromedary camels [49]. The risk of zoonotic infection was also suggested in HEV-5 (from wild boar) and HEV-8 (from a Bactrian camel) as they were successfully transmitted to cynomolgus macaques in experimental settings [112,113,114]. Besides the members of species *Orthohepevirus A*, rat HEV, which is a member of species *Orthohepevirus C*, is also capable of infecting humans, as was recently reported in both immunocompromised and immunocompetent patients [115,116,117]. With growing numbers of isolated rat HEV strains from around the world [118], as well as evidence of human infection by rat HEV [115,116,117]—in limited areas—the number of cases of human infection by rat HEV may increase in the future.

**Table 1:** Reported cross-species transmission of genus *Orthohepevirus.*

Species	Genotype	Experimental Infection	Zoonotic Potential
*Orthohepevirus A*	Human HEV-1	Non-human primate [119,120]	No
	Human HEV-2		No
	Human HEV-3	Rabbit [121]	
	Swine HEV-3	Non-human primate [17]	Yes [16,122,123,124,125,126]
	Wild boar HEV-3	Pig [127,128] Rabbit [129]	Yes [38,130,131,132] Pig [127,133]
	Deer HEV-3		Yes [39]
	Rabbit HEV-3	Pig [134,135] Non-human primate [135]	Yes [42,108]
	Human HEV-4	Pig [110]	
	Swine HEV-4	Non-human primate [109,136] Rabbit [137,138] Mongolian gerbil [139,140]	Yes [123,141,142,143]
	Wild boar HEV-4		Yes [144,145]
	Wild boar HEV-5	Non-human primate [112]	Likely
	Wild boar HEV-6		Unknown
	Dromedary camel HEV-7	Non-human primate [113]	Yes [49]
	Bactrian camel HEV-8	Non-human primate [114]	Likely
*Orthohepevirus B*	Avian HEV	Turkey [146,147]	Unlikely
*Orthohepevirus C*	Rat HEV		Yes [115,116,117]
*Orthohepevirus D*	Bat HEV		Unlikely

## 6. Mechanism of Cross-Species Transmission in HEV Infection

The key event in zoonosis is when an animal virus starts replicating in the first human subject. Here, the virus will experience the selective environment of the human body, possibly rendering viral adaptation and refinement for humans [148]. The processes responsible for cross-species transmission and the emergence of viruses in natural systems are diverse, involving ecological, evolutionary, and genetic factors. Several evolutionary mechanisms have been reported [149,150,151,152]. Among them, molecular adaptation by natural selection is an important occurrence that requires the generation and spread of beneficial mutations to increase virus fitness in a specific environment [153]. Viruses with frequent cross-species transmission events might exhibit parallel evolution (independent evolution of the same genotype or phenotype from distinct ancestors) as a result of adaptation to new host environments [153]. 

One of the efforts to demonstrate the broad range of HEV hosts was reported by Shukla et al. [154]. HEV-3 Kernow-C1 strain was semi-purified from the feces of a chronic hepatitis E patient co-infected with HIV [155]. Other than infecting human hepatoma cells (HepG2/C3A) and pig kidney cells (LLC-PK1), the fecal virus was surprisingly able to infect a variety of non-primate cells, including deer, cow, mouse, chicken, cat, dog, and rabbit cells. Meanwhile, the HEV-1 strains (Sar-55 and Akluj) used in this study successfully infected not only HepG2/C3A cells but also LLC-PK1 cells, albeit less efficiently. The HEV-3 Kernow-C1 strain was also serially passaged six times (p6). The p6 virus was shown to infect LLC-PK1 more efficiently than it did HepG2/C3A, suggesting that despite adaptation to grow in human HepG2/C3A cells, it still infected more pig cells than human cells [154].

The precise mechanism of cross-species transmission in HEV infection is not fully understood. The establishment of cross-species transmission requires the interaction of viral determinants and host determinants. Within the past decade, progress has been made regarding the viral and host determinants involved in the cross-species transmission of HEV, among which the HEV ORFs and evolutionary events have become the most studied to date (Table 2).

### 6.1. The HEV ORFs

When the ORFs were analyzed separately, ORF1 and ORF3 were demonstrated to more efficiently separate the HEV strains into anthropotropic and enzootic genotypes, suggesting their evolutionary role in host species tropism [157,158,162]. Consistent with the broad host range of enzootic HEV genotypes, the HVR of ORF1 in HEV-3 and HEV-4 is around two-fold more heterogenous than that in HEV-1 [162], and the analysis of the relative synonymous codon usage values of ORF3s showed that the HEV strains were grouped into only two clusters consisting of the HEV-1 and HEV-2 strains (anthropotropic genotypes), and a cluster of the remaining strains (HEV-3–8, enzootic genotypes) [158]. On the other hand, ORF2 was previously thought to be the viral determinant of host tropism, since it encodes the capsid (the only structural protein) that possibly binds to unknown host cell receptors [141,163,164,165].

**Figure 2:**
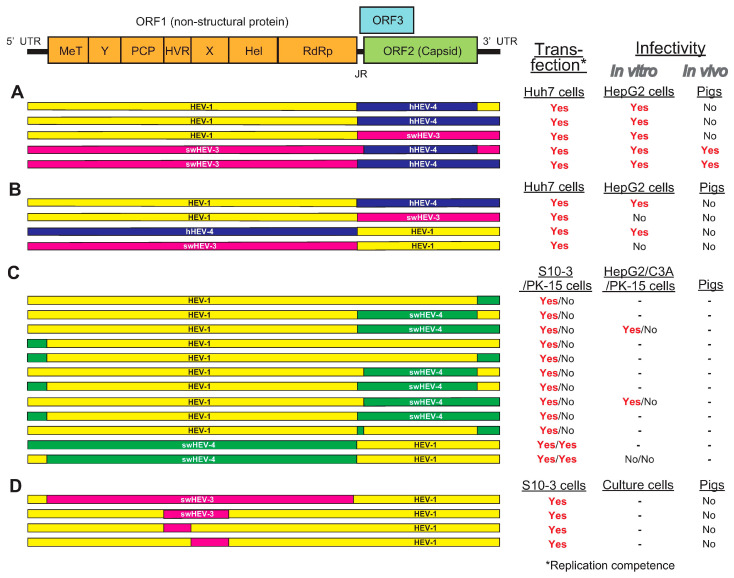
A schematic diagram of the genomic organization of HEV and its intergenotypic chimeric constructs summarized from four relevant papers. (**A**) Intergenotypic chimeric viruses where the ORF2 capsid gene—either alone or in combination—was swapped with its adjacent JR and 3′ UTR, between HEV-1 and human HEV-4, swine HEV-3 and human HEV-4, and HEV-1 and swine HEV-3 [111]. (**B**) Intergenotypic chimeric viruses where the ORF2 gene along with its JR, ORF3, and 3′ UTR were swapped between HEV-1 and human HEV-4, and between HEV-1 and swine HEV-3 [161]. (**C**) Twelve different HEV-1–swine HEV-4 intergenotypic chimeric viruses with HEV-1 as the backbone where ORF2, ORF3, ORF1, or UTRs were replaced by corresponding segments from swine HEV-4 [156]. (**D**) The genomic backbone of HEV-1 was used to construct a panel of intergenotypic chimeras in which the entire ORF1 gene or its functional domains were swapped with the corresponding regions from swine HEV-3 [168].

To unveil the viral determinant of species tropism in HEV cross-species transmission, several research groups have been utilizing the intergenotypic chimeric virus strategy. The potential role of ORF2 in HEV cross-species infection was investigated by Feagins et al. [111] and Cordoba et al. [161]. Feagins et al. [111] constructed intergenotypic chimeric viruses by swapping the ORF2 capsid gene, either alone or in combination with its adjacent junction region (JR) and the 3′ UTR, between HEV-1 and human HEV-4, swine HEV-3 and human HEV-4, and HEV-1 and swine HEV-3 (Figure 2A). Their infectivity was tested in vitro and in pigs. Three chimeric viruses containing the JR, ORF2, and 3′ UTR of swine HEV-3 or human HEV-4 in the backbone of HEV-1 failed to infect pigs. However, the other two chimeric viruses containing the ORF2 capsid gene either alone or in combination with its adjacent 5′ JR and the 3′ UTR from human HEV-4 in the backbone of swine HEV-3 were replication-competent in Huh7 cells and infectious in both HepG2/C3A cells and pigs, suggesting that the 5′ UTR and ORF1 may be involved in cross-species transmission [111].

Following this, Cordoba et al. [161] constructed four intergenotypic chimeric clones by swapping the ORF2 gene along with its JR, ORF3, and 3′ UTR between HEV-1 and HEV-4 of human origin, and HEV-1 and swine HEV-3 (Figure 2B). Only two chimeras with sequences swapped between HEV-1 and HEV-4 of human origin successfully established infection in HepG2/C3A cells. The inability of chimeras with sequences swapped between HEV-1 and swine HEV-3 to produce infectious viral particles may be due to incompatibility of viral genes from HEV-1 with those from swine HEV-3. The cDNA clone of human HEV-4 used in this study was confirmed to be capable of infecting pigs. However, none of the four chimeras were able to establish robust infection in pigs [161].

It was demonstrated that ORF2 and ORF3 are not involved in HEV cross-species infection, as evidenced by the failure of host range expansion of chimeric viruses in which the ORF2 or ORF3 gene was swapped [111,161], suggesting that other genomic regions (5′ UTR and ORF1) may play a role in determining the HEV host tropism. Chattarjee et al. [156] reported the construction of 12 different HEV-1–HEV-4 chimeric full genome clones with HEV-1 as the backbone, where the structural (ORF2 and ORF3), non-structural (ORF1), or UTRs were replaced by the corresponding segments from swine HEV-4 clone (Figure 2C). Although all chimeric clones were able to replicate in S10-3 of human hepatoma cells, only two chimeric clones (HEV-1 as the backbone where the ORF1 either alone or in combination with its 5′ UTR was swapped with those of swine HEV-4) were able to replicate in PK-15 of pig kidney cells. This shows the crucial role of the ORF1 polyprotein in crossing the species barrier at the cellular level [156].

Although the available evidence suggests that ORF1 may contribute in HEV cross-species transmission, the exact region within ORF1 that is essential in determining the host tropism is still unknown. Insertion and/or deletion in ORF1 have been demonstrated to affect not only virus replication but also host tropism [166,167]. The insertion of the host ribosomal protein sequence (RPS17) within the HVR of ORF1 led to expanded host adaptability, where the RPS17-inserted mutant was able to establish infection in cell lines of multiple animal origins in vitro [154,166], suggesting a potential role of ORF1, particularly HVR, in interspecies infection.

To determine which viral genetic element in the ORF1 is essential in determining host tropism, Tian et al. [168] utilized genomic backbone of HEV-1 which only infects humans to construct a panel of intergenotypic chimeras in which the entire ORF1 gene or its functional domains were swapped with the corresponding regions from swine HEV-3, which infects both humans and pigs (Figure 2D). Although the chimeric HEVs were replication-competent in Huh7 (S10-3) cells (a human liver cell line) and swine HEV-3 parental clone-inoculated pigs developed viremia, the RNA transcripts subsequently inoculated intrahepatically into pigs did not confer the ability to infect pigs in the chimera; this can be partially linked to the limitation of this study, where the HEV-1 backbone used here technically has low virus replication efficiency [168]. The results from this study may also suggest that—in addition to the unidentified viral genetic element within ORF1—determinants (e.g., host factors) may play a role in host tropism, either alone or in association with the viral genetic element itself. In addition, Tian et al. [168] also investigated the role of the human ribosome protein sequence S17, which expanded the host range in cultured cells [154,166], in HEV cross-species infection. The results demonstrated that the insertion of S17 in the HVR of ORF1 did not abolish HEV replication competency in vitro and that it did not expand HEV host tropism in vivo, suggesting that S17 is not a determinant factor for HEV cross-species infection in vivo [168].

### 6.2. Adaptive Evolution and the Codon Usage of HEV

The common ancestor of the modern HEV strains emerged approximately 6800 years ago, following the domestication of pigs and intensification of agriculture. Domestication caused adaptive changes in the common ancestor leading to the emergence of both human-restricted (HEV-1 and HEV-2) and zoonotic (HEV-3 and HEV-4) strains [158]. An analysis performed before the identification of the wild boar- and camel-derived genotypes (HEV-5 to HEV-8) indicated that the ancestor of all *Orthohepevirus A* was enzootic [169]. The same study also mentioned that the ancestors of HEV may have adapted to a succession of animal hosts, leading to humans. In contrast, a recent study suggested that the *Orthohepevirus A* species originated from humans and subsequently evolved into the zoonotic and human-restricted genotypes [170]. However, this study group also mentioned that their inference of an ancestral human host for extant *Orthohepevirus A* strains did not exclude the possibility that humans might have acquired HEV through cross-species transmission from other animals.

Synonymous codon usage is the non-random selection of frequently used codons. This type of selection is limited by codon bias for different genes. Determinant factors for synonymous codon usage bias include natural selection, mutational pressure, and translational efficiency, as well as compositional constraints of the mammalian genome [160,171]. It is widely known that the synonymous codon usage in viral coding sequences serves as an important mediator in viral adaptation to the host [172]. Both restricted and broad host HEV infection can provide strong natural selection leading to the shaping of the codon usage pattern of different genotypes of HEV. Viral adaptation to the host in codon usage is a long-term evolutional feedback, which is derived from the host-range of HEV infection [159].

Codon adaptation may be the essential factor in determining the viral host tropism [158]. The evolving process embodies coherent patterns of synonymous codon usage that have been established between the virus and the host. This process assists the virus in adapting to susceptible hosts and expanding the scope of zoonosis not only by specifying amino acid usage and translation dynamics but also by controlling gene expression levels [173,174].

Natural selection was identified as a major factor affecting codon usage patterns in HEV ORFs that may explain the wide range of HEV hosts, while still being influenced by mutational pressure as part of the evolution process [157,158]. In a correspondence analysis based on relative synonymous codon usage data, different HEV genotypes appeared to cluster (in particular, HEV-1, and HEV-3 and HEV-4). The analysis showed that—based on ORF1—HEV-1 is clearly separated from the other groups [160]. Considering that ORF1 encodes an essential non-structural polyprotein that controls the life cycle of HEV, these results may partially reflect the fact that HEV-1 is restricted to human hosts, while the HEV-3 and HEV-4 strains were found in various animal species with the capability of cross-species transmission [160]. Factors such as natural selection and mutational pressure enabled significant adaptation changes in the ORF1 of HEV-1 to humans, rendering ORF1 the evolutionary indicator of host speciation [157,158].

The genotype-specific codon usage pattern that results from the balance between mutational and natural selection provides an evolutionary pathway where HEV-1—with neutral evolution caused by synonymous mutations—is able to sustain its stable genetic characterization by strong natural selection. In contrast, the synonymous codon usage patterns of HEV-3 and HEV-4 may not be caused by neutral evolution. Obvious divergence of codon usage bias in a four-fold degenerate codon family was identified in HEV ORFs between HEV-1, and HEV-3 and HEV-4 [159]. The genotype-specific codon usage bias in HEV-1 is generally stronger than that of HEV-3 and HEV-4 [159]. Unlike the unique codon usage pattern of HEV-1, the HEV-3 and HEV-4 strains derived from either humans or swine have more diverse codon usage patterns in the ORFs. These can serve as evolutionary monuments and may explain the transmission from swine to human [159].

In a study by Bouquet et al. [157], although codon preferences of humans and swine are very close, a lower codon usage bias was observed for zoonotic HEV (HEV-3 and HEV-4). The codon adaptation index (CAI) in this study was calculated with the general codon usage table of humans and swine. The index is >0.5, which indicates good adaptation of HEV to its hosts; thus, it can be assumed that the gene expression of HEV of human and zoonotic genotypes is very well adapted to the translational kinetics in humans [157]. In another study, Shukla et al. [154] inoculated Sar-55 of HEV-1 and the Kernow-C1 of HEV-3 onto deer cells to study host-range restriction. Immunofluorescence staining of ORF2 protein showed that in comparison to Kernow-C1, Sar-55 was deficient in the production of ORF2 capsid protein. The introduction of the first 29 nucleotides of Kernow-C1 at the translation initiation site increased the ORF2 production of Sar-55 in deer cells, suggesting that the modulation of translation from closely spaced codons can differ significantly according to host species, and this difference may provide one mechanism for restricting the host range [154].

Synonymous codon usage patterns of HEV that are constantly modified by mutation pressure and natural selection from the host are important for the zoonotic nature of this virus. Various synonymous codon usage patterns of HEV strains may reflect the viral requirement of evolutionary adaptation to the host.

### 6.3. Interaction between HEV and the Host Cell

One of the major obstacles to interspecies virus infection is the difference between cell-receptor sequences. The primary structure of the receptor protein can affect the host susceptibility to cross-species viral infections. A virus can only adapt to a new host if the similarity between the receptor proteins present in the potential host species is high enough for them to cross the species barrier [175].

Receptor differences—either quantitative or qualitative—offer an alternative explanation for differences in host range. Supporting the hypothesis of receptor-determined host range, Shukla et al. [154] reported that the fecal Kernow-C1 p6 virus maintained a higher titer in pig cells than in human cells even though it was adapted to grow in human cells [154]. They also stated that there are 54 amino acid differences in the capsid protein between Sar-55 of HEV-1 and Kernow-C1 of HEV-3, while there are only five between the fecal and p6 Kernow-C1 strains, suggesting that the adapted virus may have retained the receptor-interacting specificity of the fecal virus [154].

Nguyen et al. [176] compared the ability of HEV-1 and HEV-3 to infect or transfect cultured human HepG2/C3A cells and swine LLC-PK cells. Immunofluorescence microscopy showed that the HEV-1 isolate infected disproportionately fewer swine cells than human cells. This study group also utilized the virus replicons of HEV-1 and HEV-3 containing the *Gaussia* luciferase gene in place of the viral ORF2 gene and showed that the luciferase expression from the HEV-1 replicon is limited and may reflect the kidney cell origin of the swine LLC-PK cells used in the study. When a kidney cell line of rhesus macaques was used for comparison, the luciferase expression patterns demonstrated that the translation of the ORF2 capsid gene of HEV-1 is inhibited in swine kidney cells in comparison to its translation in rhesus macaque kidney or human liver cells, suggesting that this virus may produce insufficient capsid protein for optimal assembly in swine cells. The severely restricted ability of both HEV-1 and its intergenotypic chimera (consisting of 450 nucleotides encoding the putative receptor-binding region of HEV-3 in place of the corresponding region of HEV-1) to infect swine kidney cells in comparison to their ability to infect human hepatoma cells supports the notion that amino acids 456 to 605 of the virus capsid protein encompass the receptor-binding region and suggests that HEV-1 may be prevented from infecting swine cells due to the absence of a suitable cellular receptor or co-receptor [176].

Although the ORF2 capsid protein was thought to play an important role in HEV attachment and entry [177], earlier studies utilizing HEV-1 as the backbone, where the ORF2 capsid gene was replaced with that of swine HEV-3 or human HEV-4, showed that HEV-1 did not acquire the ability to infect pigs [111,161]. In fact, binding to cells followed by virus entry is only the first step in virus replication. After entering the cell, the virus must interact correctly with host cellular factors to reproduce its genome and package the progeny virions [178,179,180]. Inability of the intergenotypic chimeras to infect pigs in the study by Cordoba et al. [161] showed that other than the role of viral factor itself, swine cells might lack the essential host factors required by HEV-1 to establish successful infection in pigs. Furthermore, the inability of the chimeras to infect pigs in the study by Cordoba et al. [161] may also reflect the functional importance of species-specific protein-protein interactions during HEV replication, considering that the stem-loop within the JR and 3′ UTR of HEV is known to interact with viral RdRp (and possibly with the host cellular factors) required for viral replication. In addition, the viral mechanism to counteract the host immune response can be species-specific and can partially explain the unsuccessful infection in pigs as species-specific amino acid residues that are important for evading the immune response in pigs may be absent in this setting [161]. Supporting this hypothesis, the successful replication of intergenotypic chimeras in pig kidney cells in the study by Chaterjee et al. [156] indicates that besides the importance of the interaction between ORF1 protein domains and host cell-specific factors during replication (as described above), cellular factors might play a critical role in virus establishment as a post-entry barrier.

**Figure 3:**
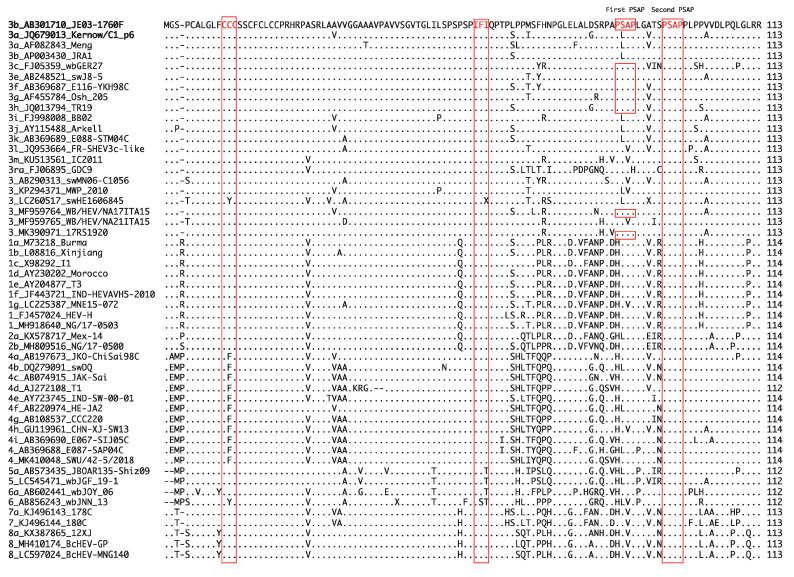
Alignment of the ORF3 amino acid sequences of *Orthohepevirus A* species showing the conserved PSAP motifs and CCC and IFI motifs. The PSAP motifs are known to be essential to the promotion of virion release [33], and the CCC and IFI motifs are reported to facilitate the virus release and ion fluxes [34].

It has been demonstrated that Sar-55 and Akluj, human strains of HEV-1, can infect LLC-PK1 swine cells [154], suggesting that human and swine HEV may share at least one cell receptor. In addition, the species-specific interactions between viral and cellular proteins may be one of the determinants of the successful replication of HEV in the host [161,181]. For instance, ORF3 is known to interact with a host cellular factor, tumor susceptibility gene 101 (Tsg101), through its PSAP motif to promote virion egress [33,182] and, thus, successful viral replication. This motif is well conserved in the HEV-1–8 strains of *Orthohepevirus A* species (Figure 3, second PSAP). In addition to this motif, JE03-1760F of HEV-3 (used in our lab) and several other isolates of HEV-3 have one additional PSAP motif located at the N-terminal, while in other HEV-3 isolates, excluding HEV-3ra, the PXXP motif is highly conserved (Figure 3, first PSAP). Besides the highly conserved PSAP motif which interacts with the host cellular factor to facilitate virion release, the highly conserved motifs of CCC and IFI of the ORF3 in *Orthohepevirus A* species (Figure 3) were recently reported to promote HEV release and ion fluxes [34]. Of note, HEV-4 has CFC in place of CCC, while HEV-5 and HEV-6 have IFT or IST in place of IFI (Figure 3). Because these motifs are highly conserved, it is possible that a host cellular factor might be the other determinant of successful viral replication.

The co-evolution of virus and the hosts often leads to species specificity. The presence of specific attachment proteins/receptors and availability of a complex pool of cellular factors required for viral replication can contribute to the host specificity [156].

### 6.4. The Host Immune Status

The immune state of the host may also be a possible determinant in HEV cross-species transmission. In most cases, humans infected with rat HEV were in an immunocompromised state [115,116]. Host adaptation after cross-species transmission is associated with rapid amino acid sequence changes of viral genes, typically those associated with receptor interactions and the evasion of innate immunity but often pervasive throughout the entire virus genome [183]. As described above, Shukla et al. [154] demonstrated the extraordinary ability of the Kernow-C1 strain to infect cells from a broad spectrum of species, ranging from rodents to primates. This probably reflects a high titer and a complex quasi-species generated during prolonged infection in an immunocompromised host. This possibility and the demonstration that HEV can acquire new information through recombination with host cell sequences leads to the conclusion that chronic HEV infection of a patient has important implications for the evolution of this emerging virus [154].

## 7. Conclusions

Knowledge on the mechanism of HEV cross-species transmission has improved drastically within the past decade. However, several important questions remain to be clarified. Based on the available data to date, ORF1 has been demonstrated as an essential viral determinant in cross-species transmission of HEV; thus, dissection of the precise domain in ORF1 that confers the ability to cross the species barrier may answer one of these important questions. In addition, as the presence and interaction of viral and host determinants are essential to enable cross-species transmission, the discovery of HEV attachment protein and its interaction with host cellular receptors will greatly help in elucidating the mechanism of HEV cross-species infection.

## Figures and Tables

**Table 2:** Possible viral and host factors involved in cross-species transmission of HEV.

	Determinants	Remarks	References
Viral factors	ORF1 (unknown domain)	Chimeric viruses where 5′ UTR, ORF1 and/or JR in the backbone of human HEV-4 were swapped with the corresponding regions of swine HEV-3 successfully infected pigs, suggesting that the 5′ UTR and ORF1 may be involved in cross-species transmission.	[111]
		Only chimeras with the swine HEV-4 ORF1 region either alone or in combination with the 5′ UTR were able to infect pig kidney cells in vitro, supporting the possible role of ORF1 in cross-species transmission.	[156]
	Adaptive evolution and codon usage	Observed bias against Sar-55 of HEV-1 ORF2 production in deer cells and its amelioration following the introduction of a short 5′ RNA sequence from the Kernow-C1 strain of HEV-3 suggests that the modulation of translation from closely spaced codons can differ significantly according to host species, and this difference may provide one mechanism for restricting the host range.	[154]
		A lower codon usage bias was observed for zoonotic HEV (HEV-3 and HEV-4). The codon adaptation index calculated with the general codon usage table for humans and swine indicates the good adaptation of HEV to its hosts. Thus, it can be assumed that the gene expression of human and zoonotic genotypes is very well adapted to the translational kinetics in humans.	[157]
		Codon adaptation may be the essential factor in determining the viral host tropism.	[158]
		The genotype-specific codon usage bias in HEV-1 is generally stronger than that of HEV-3 and HEV-4. Unlike the unique codon usage pattern of HEV-1, HEV-3 and HEV-4 strains derived from either humans or swine have more diverse codon usage patterns in ORFs.	[159]
		In a correspondence analysis based on the relative synonymous codon usage data, the different HEV genotypes appeared to cluster (in particular, HEV-1, and HEV-3 and HEV-4), and based on ORF1, HEV-1 is clearly separated from the other groups, partially reflecting that HEV-1 is restricted to human hosts, while HEV-3 and HEV-4 strains were found in various animal species and were capable of cross-species transmission.	[160]
Host factors	Host cellular factors	The inability of several intergenotypic chimeras (with HEV-1 as the genomic backbone where various genomic regions were replaced with the corresponding regions of HEV-3 or HEV-4 to infect swine either in vitro or in vivo, excluding chimeras with swapped ORF1) showed that—other than the role of the viral factor itself—swine cells might lack the essential host factors required by HEV-1 to establish successful infection in pigs. In addition, it may also reflect the functional importance of species-specific protein-protein interactions during HEV replication.	[111,156,161]
		The ability of human HEV-1 to infect pig kidney cells suggests that human and swine HEV might share at least one cell receptor.	[154]
	Host immune status	The Kernow-C1 strain of HEV-3 isolated from an immunocompromised host (HIV-infected patient) demonstrated an extraordinary ability to infect cells from a broad spectrum of species ranging from rodents to primates.	[154]
		Most cases of human infection with rat HEV involved immunocompromised individuals.	[115,116]
